# The N-terminal, WD40, and C-terminal domains of WDR-31 control ciliary localization and cooperate with ELMOD/RP2 gaps to maintain membrane compartmentalization

**DOI:** 10.55730/1300-0152.2973

**Published:** 2026-05-21

**Authors:** Sebiha ÇEVİK, Fatma GÜZEL

**Affiliations:** Rare Disease Laboratory, School of Life and Natural Sciences, Abdullah Gül University, Kayseri, Turkiye

**Keywords:** Cilia, ciliopathy, intraflagellar transport, WDR-31, ELMOD3, RP2 (retinitis pigmentosa 2)

## Abstract

**Background/aim:**

WD40 repeats are found in many ciliary proteins. Although the WD40 repeat-containing WD repeat-containing protein 31 (WDR-31) is known to regulate ciliary protein trafficking and morphology, the specific contributions of its N-terminal, WD40, and C-terminal domains to protein localization and ciliary gate integrity remain unclear. Aim of this study is to dissect the functional roles of WDR-31 domains by investigating their contributions to ciliary localization, ciliary protein trafficking, and gate integrity.

**Materials and methods:**

Using CRISPR/Cas9 technology, we generated *Caenorhabditis elegans* strains with in-frame deletions that removed the N-terminus (WDR-31(ΔN)), WD40 (WDR-31(ΔWD)), and C-terminus (WDR-31(ΔC)). We next examined the protein dynamics of wild-type WDR-31 and these variants at the ciliary base using confocal imaging and fluorescence recovery after photobleaching. Gate function was evaluated using the distribution of the periciliary marker TRAM-1, and structural ciliary abnormalities in AWB neurons were scored using confocal microscopy.

**Results:**

When the N-terminal domain was deleted, WDR-31 became less mobile at the basal body and mislocalized to the transition zone, suggesting a role in protein turnover. Furthermore, the WD40 domain is required for basal body confinement, whereas the C-terminus prevents WDR-31 from spreading into the distal axoneme. Any single domain deletion resulted in TRAM-1 leakage into the cilium in a sensitized mutant background. Remarkably, WDR-31 localization depends on IFT, the BBSome, and transition zone components; its mobility is significantly reduced in intraflagellar transport-defective mutants, supporting a model in which its steady-state distribution at the ciliary base appears to depend on intact intraflagellar transport machinery and may involve dynamic regulation of protein localization, retention, or turnover rather than being passively restricted.

**Conclusion:**

Our results indicate that the N-terminal, WD40, and C-terminal domains of WDR-31 are necessary for its dynamic positioning at the ciliary base. Together, these domains in WDR-31 are critical for maintaining TRAM-1 exclusion and periciliary membrane compartment/ciliary membrane compartmentalization.

## 1. Introduction

Cilia are ancient, tiny hair-like structures that protrude from the majority of cells, where they act like small antennae for sensing the environment (Satir and Christensen, 2007; Ishikawa and Marshall, 2011). They are built on microtubules, which provide a base and are surrounded by plasma membranes (Carvalho-Santos et al., 2011). Over the years, accumulating evidence has revealed that cilia are crucial for the development of organisms and human health (Reiter and Leroux, 2017; Anvarian et al., 2019). Specifically, cilia employ intraflagellar transport (IFT), which allows structural and signaling components to be transported in and out with extreme precision (Reiter and Leroux, 2017). A number of human diseases known as ciliopathies, such as renal issues, retinal difficulties, or skeletal deformities, arise when cilia malfunction (Szymanska and Johnson, 2012; Reiter and Leroux, 2017; Wheway et al., 2019; Park and Leroux, 2022).

IFT acts as a molecular train transporting cargo up and down the length of the cilium with the help of the BBSome complex, which serves as a sort of adaptor or cargo handler (Kozminski et al., 1993; Rosenbaum and Witman, 2002; Nachury et al., 2007; Blacque et al., 2008; Jin et al., 2010; Prevo et al., 2017). The ciliary gate structures, including the transition zone and transition fibers, are located at the base of the cilium, working as a strict checkpoint or gate (Lee and Chung, 2015). This gate determines what proteins are allowed to enter or leave the ciliary compartment (Reiter et al., 2012; Garcia-Gonzalo and Reiter, 2017; Jensen and Leroux, 2017). Over the years, many transition zone proteins that control this gate have been discovered, but we still do not fully understand all the players involved and many of the proteins in this machinery still require more study (Pir et al., 2024).

WD repeat-containing protein 31 (WDR-31) is a conserved ciliary protein containing WD40 repeats. Our previous work identified WDR-31 as a regulator of ciliary protein trafficking, functioning redundantly with GTPase-activating proteins (GAPs), including homologs of ELMOD1 and retinitis pigmentosa 2 (RP2). In *Caenorhabditis elegans*, these components cooperatively regulate IFT assembly and promote BBSome recruitment to the cilium. Disruption of this system leads to the accumulation of IFT components at the ciliary tips and impaired transport efficiency. However, the domain-specific contributions of WDR-31 to these processes and to ciliary gate function remain unknown (Cevik et al., 2023a). Although direct pathogenic variants in human *WDR31* have not yet been conclusively linked to defined ciliopathy syndromes, the conserved nature of ciliary trafficking and transition zone machinery suggests that WDR31-associated mechanisms may be broadly relevant to human cilia biology and disease. For example, in *C. elegans*, WDR-31 functions redundantly with specific GAPs, including homologs of ELMOD1, 2, and 3 and RP2. These proteins cooperatively regulate IFT assembly and the ciliary recruitment of the BBSome, thereby maintaining structural integrity and transport efficiency (Cevik et al., 2023a). RP2 and ELMOD proteins belong to a family of GAPs that regulate ARF/ARL-family small GTPases involved in membrane trafficking and ciliary transport. However, the specific contribution of individual domains to ciliary architecture and the functional mechanics of the ciliary gate remain unknown.

## 2. Materials and methods

### 2.1. Strain maintenance and generation

All strains of *C. elegans* were maintained on plates with nematode growth medium seeded with *Escherichia coli* OP50 (Brenner, 1974). The strain wdr-31 (T05A8.5) (syb1180[WDR-31::gfp]) II was used as the parental strain to generate three mutant alleles: syb1328, carrying a 476-bp deletion (C_del); syb2069, carrying a deletion of amino acids 139–261 within the WD domain (WD_del); and syb2096, carrying a deletion of amino acids 1–130 in the N-terminal region (N_del). The latter two alleles retain the C-terminal GFP tag. Genome editing and strain generation were performed by SunyBiotech (Fuzhou, China). The GFP-tagged alleles used in this study were generated by endogenous CRISPR/Cas9-mediated genome editing to preserve native expression levels and minimize overexpression-associated localization artifacts. A complete list of strains generated in this study and all primers used are provided in Supplementary Tables 1 and 2, respectively.

### 2.2. Confocal microscopy

High-resolution imaging was performed using the same confocal settings described by Cevik et al. (2023b). Briefly, live *C. elegans* hermaphrodites were immobilized on 2%–3% agarose pads with 10 mM levamisole. Z-stack images were acquired using an LSM900 confocal microscope equipped with an Airyscan 2 device controlled by ZEN 3 (Blue edition) software (ZEISS, Oberkochen, Germany). Images were processed in ZEN 3 and ImageJ (NIH, Bethesda, MD, USA) to generate maximum-intensity projections and analyze ciliary protein localization. All imaging was performed at a consistent room temperature.

### 2.3. Fluorescence recovery after photobleaching (FRAP)

FRAP experiments were carried out to examine the dynamic behavior of WDR-31::GFP variants and ELMD-1::GFP in living *C. elegans.* Nematodes were immobilized as described in Section 2.2. Photobleaching was performed using a 488-nm laser at 100% power within a circular region of interest (ROI). For basal body-associated FRAP experiments, the ROI was positioned specifically over the basal body region identified by characteristic localization patterns and nearby transition zone markers. For experiments examining ectopic ciliary localization in mutant backgrounds, the ROI was positioned within the ciliary axoneme. ROI placement was performed consistently across genotypes within each experimental set. A circular ROI was defined as the basal body in wild-type animals and the ciliary axoneme in mutant backgrounds where WDR-31 mislocalizes.

Fluorescence recovery was monitored at regular intervals for 50–100 s after bleaching. Recovery values were normalized to prebleaching intensity (set to 100%) and immediate postbleaching intensity (set to 0%). Identical photobleaching, laser power, detector gain, and image acquisition settings were used across all genotypes within each experiment to minimize technical variability.

Because several mutant backgrounds showed incomplete fluorescence recovery within the imaging window, normalized fluorescence intensity at 100 s after bleaching was used as a standardized comparative endpoint across genotypes. The mobile fraction and recovery kinetics were analyzed using the FRAP module of the ZEN 3 (Blue edition) software. The following formula for normalized intensity was used:


Normalized recovery (t)=Fpre-Fbleached/Fpost (100 s)-Fbleached

### 2.4. Ciliary gate integrity

To assess ciliary gate integrity, we used the periciliary membrane marker TRAM-1::tdTomato. TRAM-1 leakage into cilia was recorded when TRAM-1::tdTomato fluorescence extended beyond the transition zone marker MKS-2::GFP and was clearly detectable within the ciliary axoneme under identical imaging settings. The transition zone marker MKS-2::GFP was used to define the boundary between the periciliary membrane compartment (PCMC) and the ciliary compartment. For quantitative analysis, ciliary fluorescence intensity was measured only within regions distal to the transition zone. All genotypes were imaged using identical laser power, detector gain, and exposure settings. The fluorescence intensity of TRAM-1 within the ciliary axoneme was quantified using ZEN software and the Airyscan 2 (ZEISS) by measuring mean fluorescence intensity within a defined ROI encompassing the ciliary axoneme. To control for variability in transgene expression levels and imaging conditions between animals and genetic backgrounds, ciliary TRAM-1 intensity was normalized to the fluorescence intensity measured in the adjacent PCMC with the same setting. Quantification was performed across at least three independent biological replicates.

For spatial localization analysis, fluorescence intensity profiles were measured along a defined 6-μm region spanning the ciliary base and proximal cilium relative to the transition zone marker MKS-6::mCherry. The analyzed region included the area approximately 3 μm proximal to the transition zone, the transition zone region itself (~1 μm), and 2 μm distal to the transition zone. Fluorescence intensities were normalized to the maximum signal intensity within each individual cilium.

### 2.5. Ciliary morphology and phenotypic scoring

AWB ciliary morphology was visualized using the *str-1p::mCherry* reporter. Cilia were classified into four categories: wild-type – forked, wild-type – straight, extra branches (ectopic axonemal growth), and backward projections (polarity defects). For each genotype, at least 50 individual cilia were scored across three independent biological replicates.

### 2.6. Statistical analysis

All statistical analyses were performed using the R software environment (R Foundation, Vienna, Austria). Prior to applying the Welch t-test, data were assessed for normality using the Shapiro–Wilk test and for homogeneity of variance using the Levene test.

**Figure 1:**
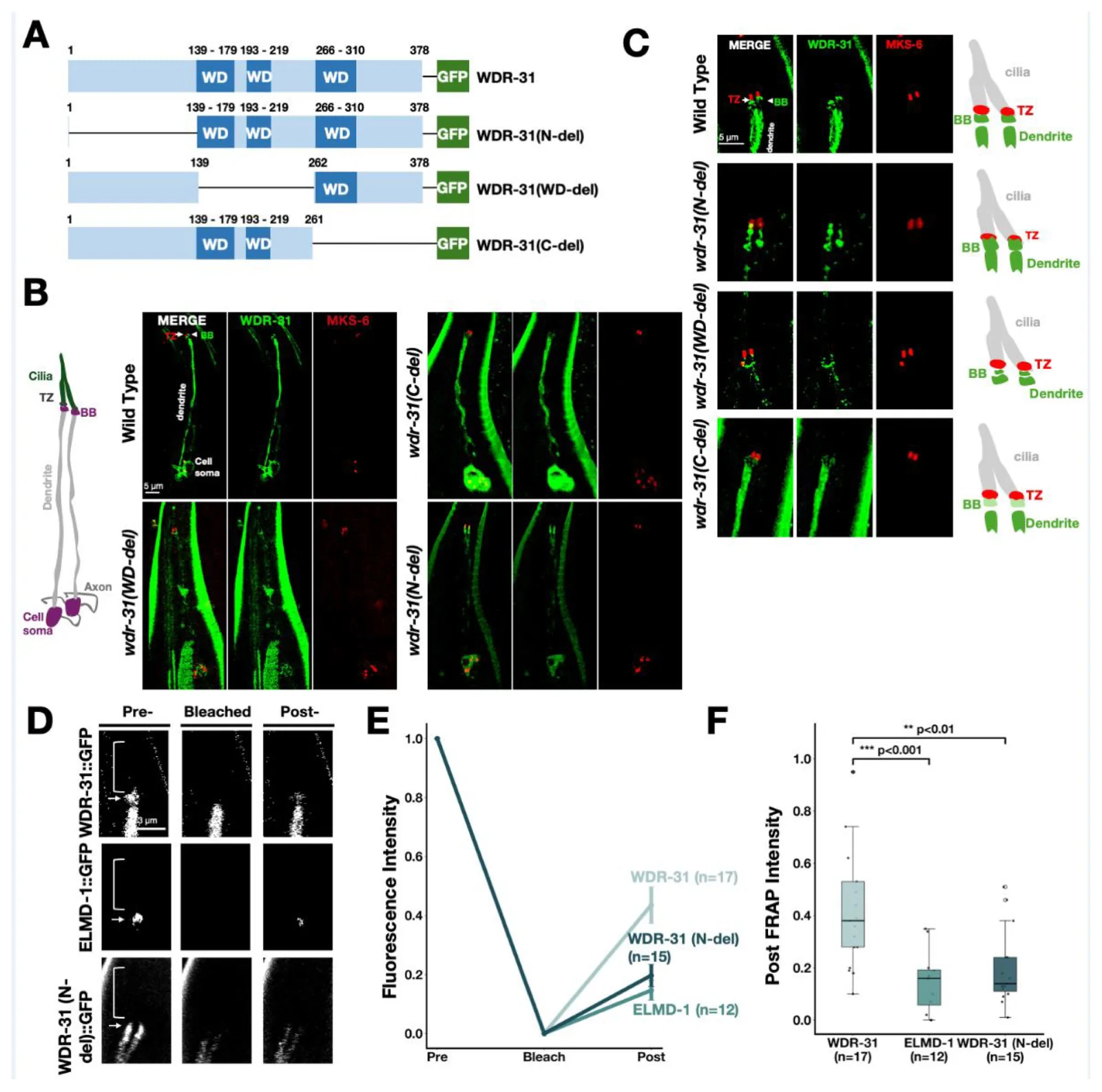
Domain analysis of WDR-31 localization and dynamics. **A)** Schematics showing the domain structure of wild-type WDR-31 and three deletion mutants: WDR-31(N-del), WDR-31(WD-del), and WDR-31(C-del). WD40 domains are highlighted in blue. **B)** Confocal images showing GFP-tagged WDR-31 variants (green) alongside transition zone marker MKS-6 (red) in *C. elegans* PHA/PHB sensory neurons. “BB” indicates the basal body, “TZ” indicates the transition zone, and the cilia, dendrite, axon, and cell soma are labeled in the schematic. Scale bar: 5 μm. **C)** Zoomed-in views of the cilium, transition zone (TZ), and basal body (BB) in each genetic background, with corresponding drawings illustrating the observed localization patterns. **D)** FRAP time series showing recovery of ELMD-1::GFP, WDR-31::GFP, and WDR-31(N-del)::GFP after photobleaching. **E)** Fluorescence recovery curves over time, showing prebleaching, bleaching, and postbleaching phases. **F)** Box plots comparing postbleaching fluorescence intensity. Statistical significance is indicated as follows: *p < 0.05, **p < 0.01, ***p < 0.001.

**Figure 2:**
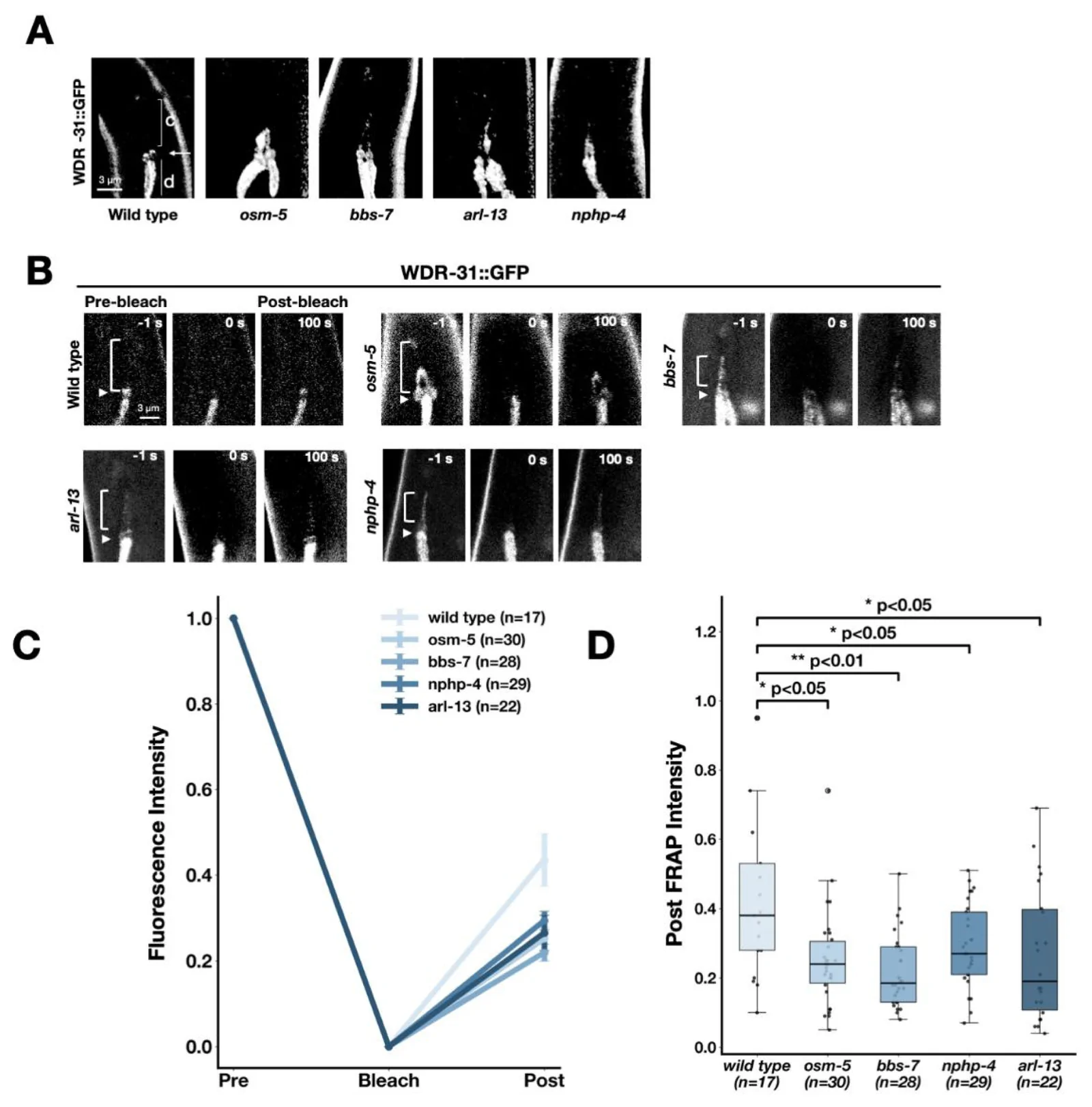
WDR-31 dynamics in cilia of mutant backgrounds. **A)** Localization of WDR-31::GFP in wild-type animals and in *osm-5*, *bbs-7*, *arl-13*, and *nphp-4* mutants. Scale bar: 3 μm. **B)** FRAP image series of WDR-31::GFP in each background at –1 s, 0 s, and 100 s after bleaching. **C)** Normalized recovery curves for each genotype over time. **D)** Quantification of postbleaching fluorescence intensity. Asterisks indicate significant differences from the wild type: *p < 0.05, **p < 0.01.

**Figure 3:**
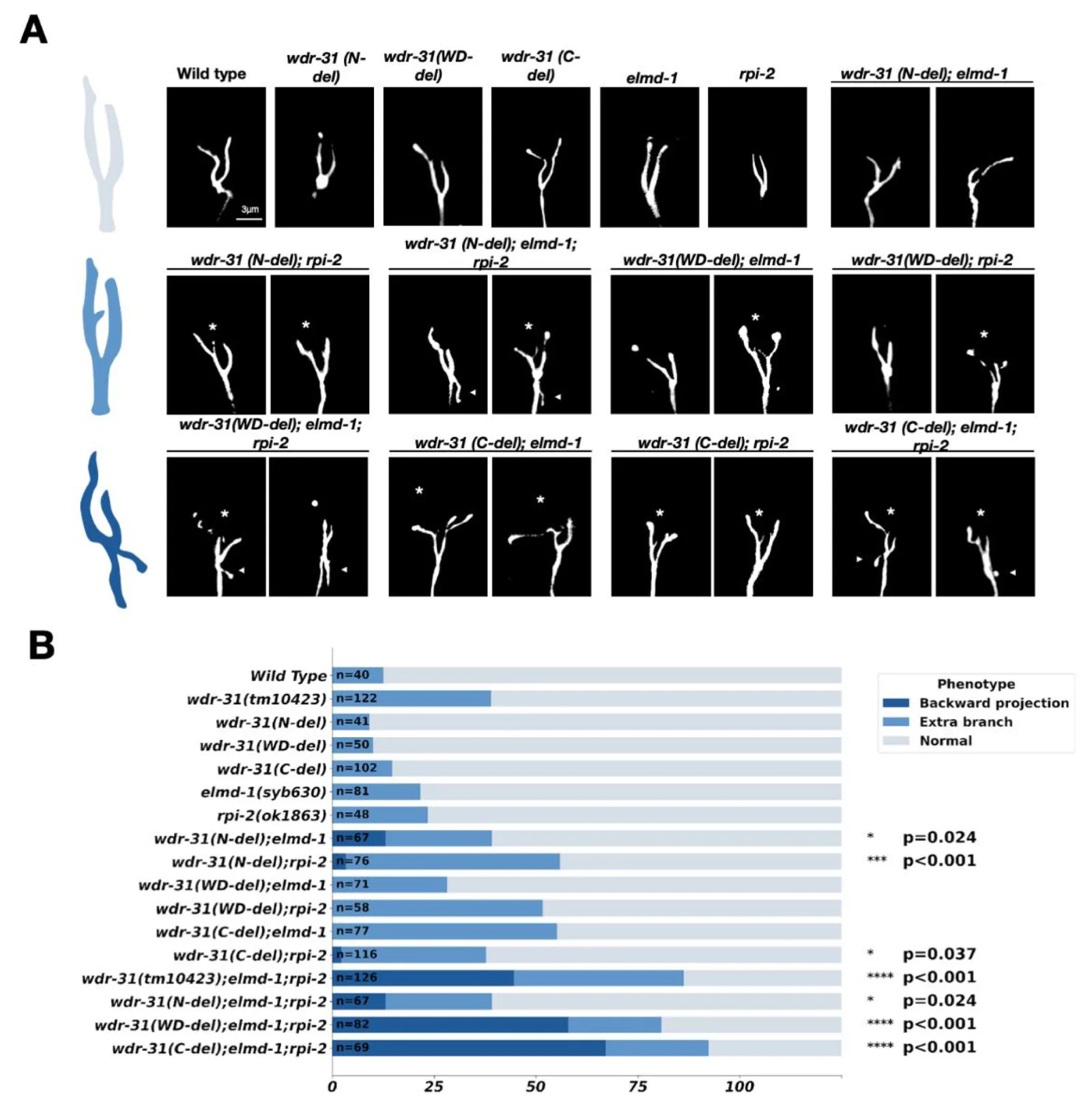
WDR-31 domains are required for restricting TRAM-1 to the PCMC. **A)** Localization of TRAM-1 (red) and transition zone marker MKS-2 (green) in various mutant backgrounds. White arrows and asterisks indicate TRAM-1::tdTomato signal extending beyond the MKS-2-marked transition zone into the ciliary axoneme. Scale bar: 1 μm. **B)** Box plots showing normalized ciliary TRAM-1 fluorescence intensity relative to the adjacent periciliary membrane compartment. Individual data points represent independent cilia measurements. Asterisks mark statistically significant differences from the wild type.

For FRAP analysis, full recovery kinetics were evaluated, including mobile fraction (F_m_) and half-time of recovery (t_1/2_), in addition to endpoint intensity measurements. For continuous data, such as the FRAP recovery measurements in Figures 1A–1F and 2A–2D and the ciliary protein localization data in Figures 3A and 3B, we used the Welch t-test to compare groups. For the FRAP experiments specifically, we compared signal intensities at the 100-s mark after photobleaching. In presenting the FRAP data in line graphs, we provided mean values with error bars representing the standard error of the mean (SEM).

**Figure 4:**
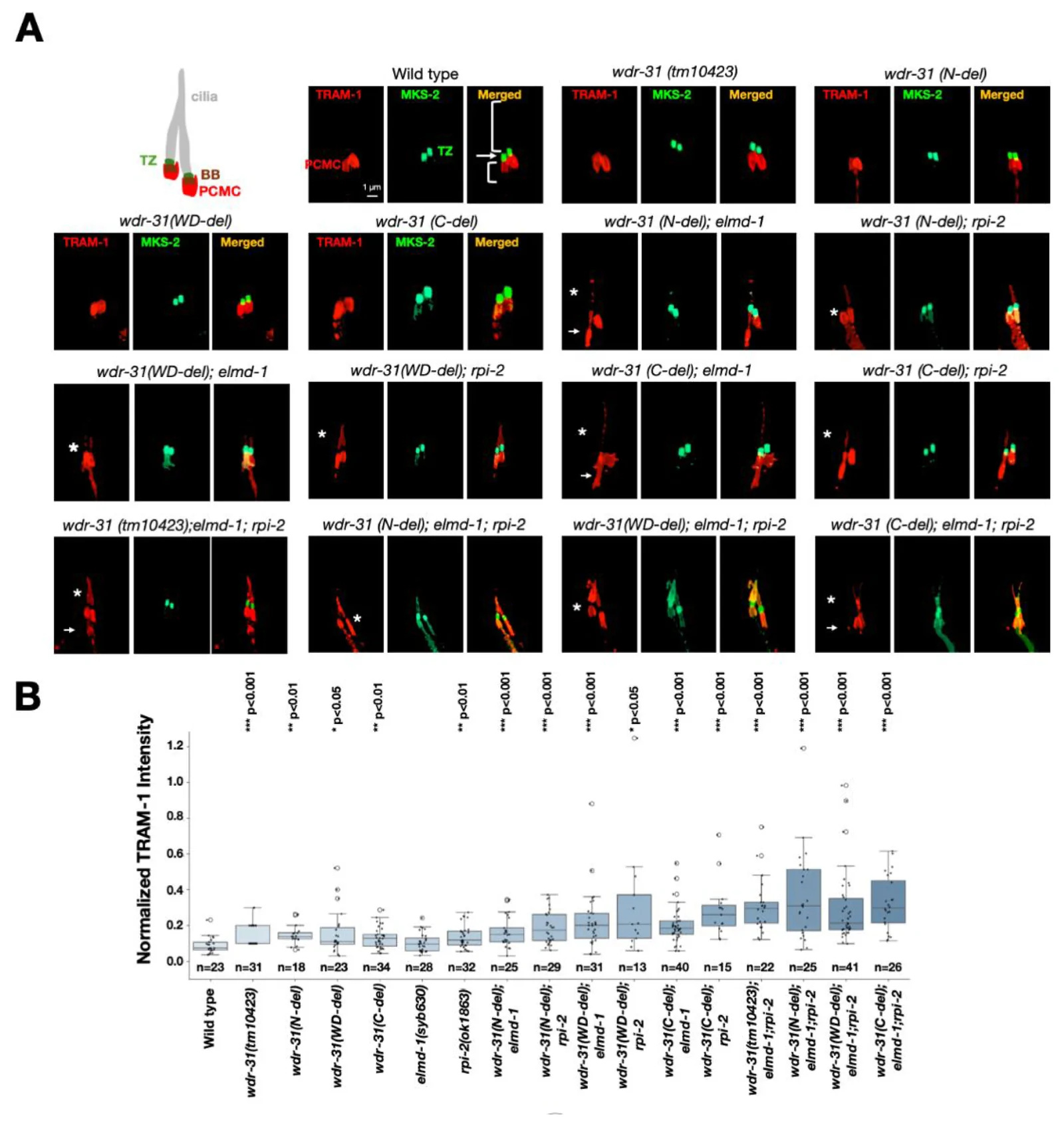
WDR-31 domains affect ciliary structure. **A)** Cilia morphology in single, double, and triple mutants lacking *wdr-31*, *elmd-1*, or *rpi-2*. Scale bar: 3 μm. **B)** Quantification of ciliary phenotypes, categorized as normal (gray), extra branch (light blue), or backward projection (dark blue). Sample sizes and p-values are shown for each genotype.

For categorical data such as the cilia morphology patterns in Figures 4A and 4B, we used the chi-square test to determine whether there were significant differences between strains. Before analyzing our larger continuous datasets (Figure 3), we filtered out the top and bottom 1% of values. For TRAM-1 leakage quantification, normalized fluorescence intensity values were compared between genotypes using the Welch t-test due to unequal variance between groups. Individual data points are displayed together with boxplots representing the median and interquartile range. For every experiment, “n” represents how many individual cilia were analyzed, and these data came from at least three independent biological replicates. We considered results statistically significant at a threshold of p < 0.05.

## 3. Results

### 3.1. WDR-31 domain architecture and requirements for ciliary localization

WDR-31 is a WD-containing ciliary protein (Cevik et al., 2023a). To investigate how different regions of WDR-31 contribute to subcellular localization, we analyzed *C. elegans* sensory neurons, focusing on AWB neurons for morphology and amphid ciliated neurons for protein localization, and we generated a series of GFP-tagged truncation mutants using CRISPR/Cas9. Specifically, we removed the N-terminal region of WDR-31, generating WDR-31(N-deletion); the two WD domains located in the middle region of the protein, generating WDR-31(WD-deletion); and the C-terminal segment, generating WDR-31(C-deletion) (Figure 1A).

Our previous work showed that WDR-31 specifically localizes to the basal body and the distal dendrite of PHA/PHB sensory neurons in *C. elegans* (Cevik et al., 2023a). We introduced wild-type WDR-31::GFP, WDR-31(WD-del)::GFP, WDR-31(C-del)::GFP, and WDR-31(N-del)::GFP into a strain carrying MKS-6, a single-copy red-tagged transition zone marker. Consistent with our previous observations, WDR-31::GFP showed minimal overlap with transition zone marker MKS-6, confirming that it predominantly localizes to the basal body and distal dendrite, forming a distinct pattern with a clear gap between the basal body and distal dendrite signals (Figures 1B and 1C; Supplementary Figure). Importantly, this localization pattern was reproducibly observed using endogenously tagged WDR-31::GFP alleles, indicating that the GFP tag does not significantly disrupt WDR-31 localization. Our analysis of truncated variants revealed domain-specific effects on this compartmentalized localization. Importantly, all truncated variants exhibited reproducible GFP signals under identical imaging conditions, suggesting that the observed localization differences are unlikely to reflect complete loss of protein expression. Deletion of the N-terminal region disrupted basal body anchoring, resulting in the mislocalization of the protein into the transition zone, as indicated by increased colocalization with transition zone marker MKS-6. Deletion of the WD domains (WD-del) did not prevent targeting to the basal body but appeared to alter protein concentration, and deletion of the C-terminal region (C-del) had minimal effect on WDR-31 localization but there was less signaling at the basal body.

### 3.2. WDR-31 ciliary exclusion and dynamic exchange are regulated by IFT, BBSome, and the transition zone gate

To further investigate how the ciliary transport machinery influences WDR-31, we examined the localization of WDR-31::GFP in several key ciliary mutant backgrounds: *osm-5* (human *IFT52*), *bbs-7* (human *BBS7*), *arl-13* (human *ARL13B*), and *nphp-4* (*NPHP4*). In wild-type animals, WDR-31 is primarily restricted to the basal body and is excluded from the ciliary axoneme. However, we observed that WDR-31 enters and accumulates within the cilia in mutants for *osm-5* (human IFT52), *bbs-7* (human *BBS7*), *arl-13* (human *ARL13B*), and *nphp-4* (*NPHP4*). This suggests that both the transition zone barrier and active transport machinery are required to maintain WDR-31 localization. While transition zone defects may permit passive entry, our FRAP analysis supports an active mechanism (Figure 2A).

To characterize the dynamics of this abnormal ciliary entry, we employed FRAP (Figure 2B). In wild-type animals, WDR-31::GFP exhibits rapid recovery at the basal body, indicating dynamic turnover. In contrast, the mislocalized pool in IFT/BBSome mutants displays a significantly reduced mobile fraction and slower recovery kinetics, consistent with loss of active transport-mediated exchange rather than simple diffusion (Figure 2C). However, the WDR-31::GFP that mislocalized to the cilia in *osm-5*, *bbs-7*, *arl-13*, and *nphp-4* mutants exhibited significantly altered mobility (Figure 2C). Notably, the recovery curves displayed varying degrees of fluorescence recovery across individual mutant backgrounds, suggesting heterogeneity in WDR-31 mobility and turnover dynamics. This variability may reflect differences in residual IFT activity, BBSome-dependent trafficking efficiency, or transition zone barrier integrity between genotypes.

Quantification of the fluorescence recovery curves and the postbleaching FRAP intensity at 100 s revealed that the mobility of WDR-31 within the cilia of these mutants was significantly reduced compared to the dynamic turnover seen at the wild-type basal body (Figure 2D). Specifically, the fluorescence recovery was markedly lower in *osm-5* and *bbs-7* mutants, suggesting that the IFT system and the BBSome contribute to the dynamic localization behavior and turnover of WDR-31.

### 3.3. WDR-31 structural domains differentially maintain ciliary morphological integrity

We next determined the effects of removing specific regions in WDR-31, including WDR-31(N-del), WDR-31(C-del), and WDR-31(WD-del), on ciliary architecture. Our previous work revealed that the combined deletion of WDR-31 and GAP proteins RP2 and ELMOD 1, 2, and 3 resulted in significant defects in IFT and cilia structures (Cevik et al., 2023a).

We first generated a range of triple mutants containing an AWB cilia marker. Wild-type AWB cilia typically have a forked appearance and straight axonemes (Figure 4A). While individual domain deletions of WDR-31 caused relatively mild structural issues, their impact became strikingly evident when combined with other genetic backgrounds. Specifically, the removal of the N-terminus (N-del) or C-terminus (C-del) primarily led to an increase in extra branches, suggesting that these domains are required to suppress ectopic axonemal growth. In contrast, removal of the WD40 repeat domain (WD-del) resulted in a higher frequency of backward projections, indicating that this domain is critical for the proper distal orientation of the cilium. Statistical analysis confirmed these observations, showing that the combination of individual domain deletions with *rpi-2* and *elmd-1* significantly increased structural abnormalities (p < 0.001) to levels similar to that of full *wdr-31* (*tm10423*) knockout (Figure 4B**)**.

### 3.4. WDR-31 structural domains are required for ciliary gate integrity

The transition zone acts as a gatekeeping barrier, keeping periciliary membrane proteins like TRAM-1 out of the cilium. TRAM-1 remains tightly confined to the periciliary compartment, never entering the cilium. Previous studies used TRAM-1 mislocalization to cilia as a readout of gate integrity (Figure 3A) (Williams et al., 2011; Jensen et al., 2015; Palander et al., 2017; Cevik et al., 2023a). Thus, we assessed the effects of specific structural domains of WDR-31 on transition zone gatekeeping utilizing the precise location of PCMC marker TRAM-1. As expected, deleting individual WDR-31 domains caused minimal leakage of TRAM-1 into the cilium (Figure 3A). However, combining any single domain deletion with loss of *elmd-1* and *rpi-2* resulted in dramatic and quantitatively significant TRAM-1 leakage into the ciliary axoneme (p < 0.001), as determined by normalized ciliary-to-PCMC fluorescence intensity measurements. Representative images revealed this gatekeeping failure, showing TRAM-1 (red) extending past transition zone marker MKS-2 (green) (Figures 3A and 3B). These findings demonstrate that all three WDR-31 domains are essential for robust ciliary gate maintenance, with partial structural compromise sufficient to disrupt membrane protein exclusion in sensitized contexts.

## 4. Discussion

While it is currently unknown if WDR-31 has an essential function in human development, research involving zebrafish has begun to clarify its role. Zebrafish *wdr-31* knockouts show decreased cilia number in the otic vesicle and heart edema (Cevik et al., 2023a). This relatively mild phenotype aligns with what we see in *C. elegans*: removing *wdr-31* alone causes little overt disruption (Cevik et al., 2023a). WDR-31 only becomes essential for cilia biology when GAP proteins RP2 and ELMOD1, 2, and 3 are missing. Even though WDR-31 is not currently linked to any human disease, we used CRISPR/Cas9 technology to determine the domains critical for its function in cilia biology.

Our domain analysis builds on the model proposed by Cevik et al. (2023a), who showed that WDR-31 functions redundantly with GAP proteins RP-2 and ELMD-1 to regulate IFT and BBSome trafficking. We found that triple mutants lacking all three genes exhibit dramatic phenotypes: IFT components accumulate at the ciliary tips, fewer IFT/BBSome particles travel along the cilia, and nonciliary proteins like TRAM-1 leak into the axoneme. Because the present analysis relies on TRAM-1 as a representative PCMC marker, we interpret these data as evidence for impaired TRAM-1 exclusion and defective PCMC/ciliary compartmentalization. Future analysis using additional PCMC or ciliary membrane proteins will be important for determining whether WDR-31 domain loss causes broad gate disruption or affects specific membrane-associated cargos.

Our data extend the previous findings by showing that even partial loss of WDR-31 involving the removal of any single domain is enough to trigger these same defects when combined with *rpi-2* and *elmd-1* deletions. Interestingly, the FRAP recovery profiles also showed variable recovery kinetics across mutant backgrounds, indicating that WDR-31 dynamics are not uniform across defective cilia. This heterogeneity may arise from differences in the remaining activity of IFT machinery, BBSome-dependent transport, or local transition zone organization. The partial recovery observed in some mutants suggests that WDR-31 mobility is not completely abolished; instead, it is dynamically constrained depending on the severity of ciliary transport disruption.

The N-terminus, WD40 domain, and C-terminus are each required for full gatekeeping function, but their contributions appear qualitatively different. Our findings demonstrate that the localization of WDR-31 at the ciliary base is a dynamically maintained state rather than a passive property. The N-terminus anchors the protein at the basal body, the WD40 domain ensures proper concentration and spatial organization, and the C-terminus suppresses ectopic axonemal expansion. Mislocalization disrupts this spatial control, leading to failure of the ciliary gate and inappropriate entry of nonciliary proteins such as TRAM-1.

Current models propose that the transition zone functions not simply as a static diffusion barrier but rather as a dynamic gating interface that coordinates selective protein entry, membrane compartmentalization, and trafficking into the cilium. In this framework, the transition zone cooperates with IFT- and BBSome-mediated transport systems to regulate both soluble and membrane-associated cargos. Our findings are consistent with such modeling, as disruption of WDR-31 domains alters protein localization, affects FRAP recovery behavior, and permits abnormal entry of the PCMC marker TRAM-1 into the ciliary compartment. Furthermore, the strong genetic interactions observed with IFT, BBSome, and transition zone-associated components support a role for WDR-31 in coordinated transport and compartmentalization at the ciliary base. Although reduced FRAP recovery in IFT-defective backgrounds is consistent with altered WDR-31 dynamics, our data do not directly distinguish between active transport-mediated positioning and alternative mechanisms such as altered retention, stability, diffusion, or compartmental confinement.

An important mechanistic aspect of this pathway may involve ARF/ARL-family small GTPase regulation. RP2 and ELMOD proteins function as GAPs, which promote the hydrolysis of GTP-bound ARF/ARL-family GTPases into their inactive GDP-bound state. ARL-family GTPases are well-established regulators of ciliary trafficking, membrane identity, and BBSome-dependent transport. Thus, the cooperative relationship among WDR-31, RP2, and ELMD-1 may reflect a broader regulatory network that coordinates GTPase cycling with ciliary gate maintenance and intraflagellar transport dynamics. Defects in this regulatory system could alter the spatial organization of transport machinery at the ciliary base, thereby contributing to abnormal WDR-31 localization, altered FRAP dynamics, and defective exclusion of membrane-associated proteins such as TRAM-1.

The proposed interaction between WDR-31 and ELMOD/RP2 GAP proteins is currently based on genetic evidence. Ongoing TurboID proximity labeling experiments aim to identify domain-specific interactors and refine this model. Understanding which proteins interact with the N-terminal, WD40, and C-terminal domains of WDR-31 may help clarify how these regions contribute to retention of WDR-31 at the basal body, maintenance of its proper localization, and suppression of ectopic branching. The mechanisms described here may also have broader relevance to human ciliopathies. Defects in IFT, BBSome trafficking, transition zone organization, and membrane compartmentalization are central pathogenic features of many human ciliopathies, including Joubert syndrome, Bardet–Biedl syndrome, nephronophthisis, and Meckel syndrome. Our findings place WDR-31 within this conserved transport-gating network, particularly through its functional interactions with IFT machinery, BBSome-associated pathways, and RP2/ELMOD GAP proteins. The observed defects in ciliary protein dynamics and membrane compartmentalization suggest that disruption of WDR31-associated pathways could contribute to disease mechanisms involving defective ciliary trafficking or transition zone integrity. Given the strong evolutionary conservation of ciliary architecture, further investigation of human WDR31 variants and interacting partners may provide insight into currently unresolved ciliopathy cases.

## Supplementary Materials

Supplementary Figure Loss of N-terminal, WD-repeat, or C-terminal regions of WDR-31 shifts its localization away from the basal body in *C. elegans* sensory cilia. Colocalization analysis of WDR-31::GFP (green) and MKS-6::mCherry (red, transition-zone marker) along the proximal–distal axis of the cilium. Fluorescence intensity was measured in 2 μm bins from the basal body (0–2 μm) to the ciliary tip (4–6 μm) and normalized within each cilium. Panels show the full-length protein and three deletion variants: (A) WDR-31 (full-length), n = 9 cilia; (B) WDR-31 (N-del), N-terminal deletion, n = 9; (C) WDR-31 (WD-del), deletion of the WD-repeat region, n = 7; (D) WDR-31 (C-del), C-terminal deletion, n = 12. In each panel, the colored line with circles represents the mean WDR-31::GFP signal (±SEM, error bars) and the red shaded area with dashed outline represents the mean MKS-6::mCherry signal, which marks the transition zone at 3–4 μm in all conditions. Inverted triangles (▾) indicate the bin containing the peak intensity for each channel, and the curved arrow denotes the offset between the MKS-6 and WDR-31 peaks. Full-length WDR-31 peaks at the basal body (0–1 μm bin, panel A), where its signal does not overlap with MKS-6. In contrast, all three deletion variants exhibit a marked depletion of WDR-31 from the basal body and a redistribution toward the proximal cilium, with the peak shifted to the 2–3 μm bin immediately basal to the MKS-6-marked transition zone (panels B–D). The MKS-6 distribution is unchanged across all four genetic backgrounds, confirming that transition-zone architecture is intact and that the observed redistribution reflects a specific defect in WDR-31 targeting. Together, these data indicate that the N-terminus, WD-repeat domain, and C-terminus each contribute to the basal-body localization of WDR-31.

**Supplementary Table 1:** Caenorhabditis elegans strains used in this study.

#	Strain name	Genotype	Study
1	OIK328	*wdr-31(T05A8.5)(syb1180[wdr-31::gfp]) II.*	Cevik et al. 2023a
2	PHX1328	*wdr-31(T05A8.5)(syb1328[wdr-31::C_del( 476 bd deletion) ::gfp]) II.*	This study was generated by SunyBiotech
3	PHX2069	*wdr-31(T05A8.5)(syb2069[wdr-31::WD_del( 139 -261 aa deletion) ::gfp]) II.*	This study was generated by SunyBiotech
4	PHX2096	*wdr-31(T05A8.5)(syb2096[wdr-31::N_del( 1–130 aa_deletion) ::gfp]) II.*	This study was generated by SunyBiotech
5	OIK1092	*wdr-31(T05A8.5)(syb1180[wdr-31::gfp]) II.. ; vuaSi21[pBP39; Pmks-6::mks-6::mCherry; cb-unc-119(+)]*	This study
6	OIK1803	*wdr-31(T05A8.5)(syb1328[wdr-31::C_del( 476 bd deletion) ::gfp]) II. ; vuaSi21[pBP39; Pmks-6::mks-6::mCherry; cb-unc-119(+)]*	This study
7	OIK786	*wdr-31(T05A8.5)(syb2069[wdr-31::WD_del( 139 -261 aa deletion) ::gfp]) II. ; vuaSi21[pBP39; Pmks-6::mks-6::mCherry; cb-unc-119(+)]*	This study
8	OIK1801	*wdr-31(T05A8.5)(syb2096[wdr-31::N_del( 1–130 aa_deletion) ::gfp]) II.. ; vuaSi21[pBP39; Pmks-6::mks-6::mCherry; cb-unc-119(+)]*	This study
9	FX925	*nphp-4(tm925)*	CGC
10	VC1104	*arl-13 (gk513)*	CGC
11	RB1268	*bbs-7(ok1351)*	CGC
12	PR813	*osm-5(p813)*	CGC
13	OIK1791	*nphp-4(tm925); wdr-31(T05A8.5)(sy11180[wdr-31::gfp]) II.*	This study
14	OIK1792	*arl-13 (gk513);wdr-31(T05A8.5)(sy11180[wdr-31::gfp]) II.*	This study
15	OIK1793	*bbs-7(ok1351);wdr-31(T05A8.5)(sy11180[wdr-31::gfp]) II.*	This study
16	OIK1794	*osm-5(p813);wdr-31(T05A8.5)(sy11180[wdr-31::gfp]) II.*	This study
17	FX30333	*wdr-31 (tm10423)*	Cevik et al., 2023a
18	OIK1802	*wdr-31 (tm10423); oyIs65[str-1p::mcherry]*	Cevik et al., 2023a
19	OIK474	*wdr-31 (tm10423); N2;Ex [mks-2:gfp+tram-1:td tomato+ rol-6]*	Cevik et al., 2023a
20	OIK557	*wdr-31 (tm10423);elmd-1(syb630);rpi-2(ok1863); oyIs65[str-1p::mcherry]*	Cevik et al., 2023a
21	OIK560	*wdr-31 (tm10423); elmd-1(syb630);rpi-2(ok1863); N2;Ex [mks-2:gfp+tram-1:td tomato+ rol-6]*	Cevik et al., 2023a
22	PHX630	*elmd-1(syb630)*	Cevik et al., 2023a
23	OIK1685	*elmd-1(syb630); oyIs65[str-1p::mcherry]*	Cevik et al., 2023a
24	OIK475	*elmd-1(syb630); N2;Ex [mks-2:gfp+tram-1:td tomato+ rol-6]*	Cevik et al., 2023a
25	Rb1550	*rpi-2(ok1863)*	Cevik et al., 2023a
26	OIK571	*rpi-2(ok1863); oyIs65[str-1p::mcherry]*	Cevik et al 2023a
27	OIK485	*rpi-2(ok1863); N2;Ex [mks-2:gfp+tram-1:td tomato+ rol-6]*	Cevik et al., 2023a
28	OIK1805	*wdr-31(T05A8.5)(syb2096[wdr-31::N_del( 1–130 aa_deletion) ::gfp]) II.;oyIs65[str-1p::mcherry]*	This study
29	OIK1808	*wdr-31(T05A8.5)(syb2096[wdr-31::N_del( 1–130 aa_deletion) ::gfp]) II.;elmd-1(syb630); oyIs65[str-1p::mcherry]*	This study
30	OIK1806	*wdr-31(T05A8.5)(syb2096[wdr-31::N_del( 1–130 aa_deletion) ::gfp]) II.;rpi-2(ok1863); oyIs65[str-1p::mcherry]*	This study
31	OIK1807	*wdr-31(T05A8.5)(syb2096[wdr-31::N_del( 1–130 aa_deletion) ::gfp]) II.;elmd-1(syb630);rpi-2(ok1863); oyIs65[str-1p::mcherry]*	This study
32	OIK1795	*wdr-31(T05A8.5)(syb2096[wdr-31::N_del( 1–130 aa_deletion) ::gfp]) II.; N2;Ex [mks-2:gfp+tram-1:td tomato+ rol-6]*	This study
33	OIK1809	*wdr-31(T05A8.5)(syb2096[wdr-31::N_del( 1–130 aa_deletion) ::gfp]) II.;elmd-1(syb630); N2;Ex [mks-2:gfp+tram-1:td tomato+ rol-6]*	This study
34	OIK1796	*wdr-31(T05A8.5)(syb2096[wdr-31::N_del( 1–130 aa_deletion) ::gfp]) II.;rpi-2(ok1863); N2;Ex [mks-2:gfp+tram-1:td tomato+ rol-6]*	This study
35	OIK882	*wdr-31(T05A8.5)(syb2096[wdr-31::N_del( 1–130 aa_deletion) ::gfp]) II.;elmd-1(syb630);rpi-2(ok1863); N2;Ex [mks-2:gfp+tram-1:td tomato+ rol-6]*	This study
36	OIK588	*wdr-31(T05A8.5)(syb1328[wdr-31::C_del( 476 bd deletion) ::gfp]) II. ;oyIs65[str-1p::mcherry]*	This study
37	OIK590	*wdr-31(T05A8.5)(syb1328[wdr-31::C_del( 476 bd deletion) ::gfp]) II.;elmd-1(syb630); oyIs65[str-1p::mcherry]*	This study
38	OIK1686	*wdr-31 (C-del);rpi-2(ok1863); oyIs65[str-1p::mcherry]*	This study
39	OIK891	*wdr-31(T05A8.5)(syb1328[wdr-31::C_del( 476 bd deletion) ::gfp]) II.;elmd-1(syb630);rpi-2(ok1863); oyIs65[str-1p::mcherry]*	This study
40	OIK785	*wdr-31(T05A8.5)(syb1328[wdr-31::C_del( 476 bd deletion) ::gfp]) II.; N2;Ex [mks-2:gfp+tram-1:td tomato+ rol-6]*	This study
41	OIK783	*wdr-31(T05A8.5)(syb1328[wdr-31::C_del( 476 bd deletion) ::gfp]) II.;elmd-1(syb630); N2;Ex [mks-2:gfp+tram-1:td tomato+ rol-6]*	This study
42	OIK784	*wdr-31(T05A8.5)(syb1328[wdr-31::C_del( 476 bd deletion) ::gfp]) II.;rpi-2(ok1863); N2;Ex [mks-2:gfp+tram-1:td tomato+ rol-6]*	This study
43	OIK782	*wdr-31(T05A8.5)(syb1328[wdr-31::C_del( 476 bd deletion) ::gfp]) II.;elmd-1(syb630);rpi-2(ok1863); N2;Ex [mks-2:gfp+tram-1:td tomato+ rol-6]*	This study
44	OIK1681	*wdr-31(T05A8.5)(syb2069[wdr-31::WD_del( 139 -261 aa deletion) ::gfp]) II.;oyIs65[str-1p::mcherry]*	This study
45	OIK1682	*wdr-31(T05A8.5)(syb2069[wdr-31::WD_del( 139 -261 aa deletion) ::gfp]) II.;elmd-1(syb630); oyIs65[str-1p::mcherry]*	This study
46	OIK1555	*wdr-31(T05A8.5)(syb2069[wdr-31::WD_del( 139 -261 aa deletion) ::gfp]) II.;rpi-2(ok1863); oyIs65[str-1p::mcherry]*	This study
47	OIK881	*wdr-31(T05A8.5)(syb2069[wdr-31::WD_del( 139 -261 aa deletion) ::gfp]) II.;elmd-1(syb630);rpi-2(ok1863); oyIs65[str-1p::mcherry]*	This study
48	OIK781	*wdr-31(T05A8.5)(syb2069[wdr-31::WD_del( 139 -261 aa deletion) ::gfp]) II.; N2;Ex [mks-2:gfp+tram-1:td tomato+ rol-6]*	This study
49	OIK779	*wdr-31(T05A8.5)(syb2069[wdr-31::WD_del( 139 -261 aa deletion) ::gfp]) II.;elmd-1(syb630); N2;Ex [mks-2:gfp+tram-1:td tomato+ rol-6]*	This study
50	OIK780	*wdr-31(T05A8.5)(syb2069[wdr-31::WD_del( 139–261 aa deletion) ::gfp]) II.;rpi-2(ok1863[mks-2:gfp+tram-1:td tomato+ rol-6]*	This study
51	OIK778	*wdr-31(T05A8.5)(syb2069[wdr-31::WD_del( 139–261 aa deletion) ::gfp]) II.;elmd-1(syb630);rpi-2(ok1863); N2;Ex [mks-2:gfp+tram-1:td tomato+ rol-6]*	This study
52	OIK1754	*N2;Ex [mks-2:gfp+tram-1:td tomato+ rol-6]*	
53	OIK1052	*oyls65[str-1p::mcherry]*	Sengupta Lab

**Supplementary Table 2:** Primer sequences used for genotyping the C. elegans strains used in this study.

Oligonucleotides			
	Genotype Name	Forward Primer (5′>3′)	Reverse Primer (5′>3′)
1	*wdr-31(T05A8.5)(syb2069[wdr-31::WD_del (139 -261 aa deletion) ::gfp]) II.*	CTGACAAACACCGACAGTGG	CTTGAAGATTATCCGCTTCC
2	*wdr-31(T05A8.5)(syb2096[wdr-31::N_del (1-130 aa_deletion) ::gfp]) II.*	ccgagcacaagagaaggctc	TCAAACTCGCAGAACATCTG
3	*wdr-31 (tm10423) II.*	gtggtaccatagagctactg	CACCCCGAACTTGTGTCCATC
4	*rpi-2(ok1863) IV.*	gagacgcagacatctcatctg	caggtcgttctcggacatcac
5	*elmd-1(syb630)III.*	gtggatgggtatcacagtgg	gttcgcttacacacgctttc
6	*arl-13(gk513)*	gcatatggcgtcacaatgacc	cacaactccaacaaaaatgactcg
7	*bbs-7(ok1351)*	ctacagtacccccacagtgctc	cgttccacgtcaccagatacc
8	*nphp-4(tm925)*	ccagcagcttgaaatagcag	gactgagaacattcgataccag
